# Simplified and Highly-reliable automated production of [^18^F]FSPG for clinical studies

**DOI:** 10.21203/rs.3.rs-3031030/v1

**Published:** 2023-06-26

**Authors:** Mai Lin, Robert T. Ta, H. Charles Manning

**Affiliations:** The University of Texas MD Anderson Cancer Center; The University of Texas MD Anderson Cancer Center; The University of Texas MD Anderson Cancer Center

## Abstract

**Background:**

(S)-4-(3-^18^F-Fluoropropyl)-L-Glutamic Acid ([^18^F]FSPG) is a positron emission tomography (PET) tracer that specifically targets the cystine/glutamate antiporter (xc-), which is frequently overexpressed in cancer and several neurological disorders. Pilot studies examining the dosimetry and biodistribution of ([^18^F]FSPG in healthy volunteers and tumor detection in patients with non-small cell lung cancer, hepatocellular carcinoma, and brain tumors showed promising results. In particular, low background uptake in the brain, lung, liver, and bowel was observed that further leads to excellent imaging contrasts of [^18^F]FSPG PET. However, reliable production-scale cGMP-compliant automated procedures for [^18^F]FSPG production are still lacking to further increase the utility and clinical adoption of this radiotracer. Herein, we report the optimized automated approaches to produce [^18^F]FSPG through two commercially available radiosynthesizers capable of supporting centralized and large-scale production for clinical use.

**Results:**

Starting with activity levels of 60–85 GBq, the fully-automated process to produce [^18^F]FSPG took less than 45 minutes with average radiochemical yields of 22.56 ± 0.97% and 30.82 ± 1.60% (non-decay corrected) using TRACERlab^™^ FXFN and FASTlab^™^, respectively. The radiochemical purities were > 95% and the formulated [^18^F]FSPG solution was determined to be sterile and colorless with the pH of 6.5–7.5. No radiolysis of the product was observed up to 8 hours after final batch formulation.

**Conclusions:**

In summary, cGMP-compliant radiosyntheses and quality control of [^18^F]FSPG have been established on two commercially available synthesizers leveraging high activity concentration and radiochemical purity. While the clinical trials using [^18^F]FSPG PET are currently underway, the automated approaches reported herein will accelerate the clinical adoption of this radiotracer and warrant centralized and large-scale production of [^18^F]FSPG.

## Background

Positron emission tomography (PET) is both a medical and research tool used in pre-clinical and clinical settings. It is widely applied in the imaging of tumors and the search for metastases within the field of clinical oncology, and for the clinical diagnosis of certain neurological disorders. Among all radiopharmaceuticals for PET imaging, the glucose analogue [^18^F]fluorodeoxyglucose ([^18^F]FDG) is the most heavily used to date in oncological applications. However, there are clinical gaps in the effectiveness of [^18^F]FDG PET, and increasingly, PET tracers targeting very specific molecular and even ‘druggable’ pathways are required. As a result, there is a great demand for additional radiopharmaceuticals with unique targeting capability for molecular imaging.

Amino acids play a variety of critical roles in many cellular functions. Due to an accelerated growth rate in cancer cells, the demand for amino acids is elevated as well. Consequently, imaging radiopharmaceuticals based on radiolabeled amino acid analogues have long been appreciated to provide information regarding protein metabolism of malignant cells. Recently, a glutamate-based tracer, (S)-4-(3-[^18^F]fluoropropyl)-L-glutamic acid ([^18^F]FSPG), has started to receive great attention as it has been shown to specifically target the cystine/glutamate antiporter (xc^−^). The xc^−^ antiporter is a plasma membrane transporter mediating cellular uptake of cystine in exchange for intracellular glutamate with an equal stoichiometry ratio (Lo, Ling, Wang, & Gout, 2008). Although cysteine plays important roles in protein synthesis and in maintaining redox balance, *de novo* biosynthesis or a catabolic supply of cysteine is not sufficient to meet the high demand for antioxidant synthesis under conditions of oxidative stress such as in cancer and neurological disorders, which drives xc- activity to ensure cysteine supply (Combs & DeNicola, 2019; Koppula, Zhang, Zhuang, & Gan, 2018; Lewerenz et al., 2013). More recently, Lei et al. discovered that xc^−^ antiporter activity promotes radioresistance largely through inhibiting ferroptosis, a form of regulated cell death induced by lipid peroxidation (Lei et al., 2020). These authors found that the xc^−^ antiporter is a target of BRCA1-associated protein 1 (BAP1) and that BAP1 promotes ferroptosis through repressing xc^−^ antiporter expression, resulting in tumor suppression (Zhang et al., 2018). Indeed, BAP1 deletions and loss of function mutations are common in many cancers (Carbone et al., 2020; Han, Purwin, & Aplin, 2021; Murali, Wiesner, & Scolyer, 2013; Yan et al., 2022), and in these tumors, xc^−^ activity would be likely to provide extracellular cystine and tumor protection. In this way, [^18^F]FSPG PET could provide an indirect measure of BAP1 activity, which could result in a better understanding of therapeutic efficacy during the course of various treatments. Recent clinical studies examining dosimetry and biodistribution of [^18^F]FSPG in healthy volunteers (Mosci et al., 2016; Smolarz et al., 2013) and tumor detection in patients with non-small cell lung cancer (NSCLC), hepatocellular carcinoma, and brain tumors have shown promising results (Baek et al., 2012; Kavanaugh et al., 2016; Magarik, Walker, Gilbert, Manning, & Massion, 2018; Mittra et al., 2016; Paez et al., 2022; S. Park et al., 2017; Wardak et al., 2022). Of note, the low uptake in the brain, lung, liver, and bowel renders [^18^F]FSPG an excellent imaging agent characterized by high tumor-to-background ratios (S. Y. Park et al., 2020).

Producing radiotracers with highly consistent radiochemical yield and quality is required to bring promising radiotracers from bench to bedside. Although several groups have described automated procedures to produce [^18^F]FSPG, previous reports were focused on small-scale production (less than 400 MBq of the final product) and with complex modifications of commercially available cartridges (Brown, Soloviev, & Lewis, 2023; Edwards, Greenwood, McRobbie, Khan, & Witney, 2021; Kavanaugh et al., 2016; Koglin et al., 2011; McCormick et al., 2019; Mittra et al., 2016; Shih et al., 2020), making it challenging to directly implement at a manufacturing facility. Here, we report a detailed and highly robust technical protocol to produce the large-scale, cGMP-compliant radiosynthesis of [^18^F]FSPG using GE TRACERlab^™^ FXFN and FASTlab^™^, the most widely used synthesis modules for routine clinical radiopharmaceutical production. The protocol detailed herein highlights numerous improvements over prior reports, addressing critical limitations, and can be easily followed by anyone skilled in the art and equipped with common resources.

## Materials and Methods

### General

**Figure 1:**
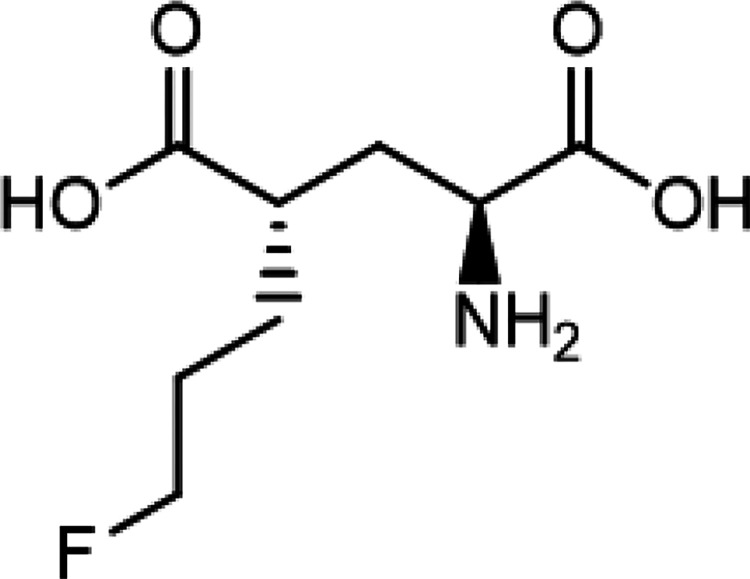
Chemical structure of (4S)-4-(3-fluoropropyl)L-glutamate as the reference standard of [^18^F]FSPG.

**Figure 2:**
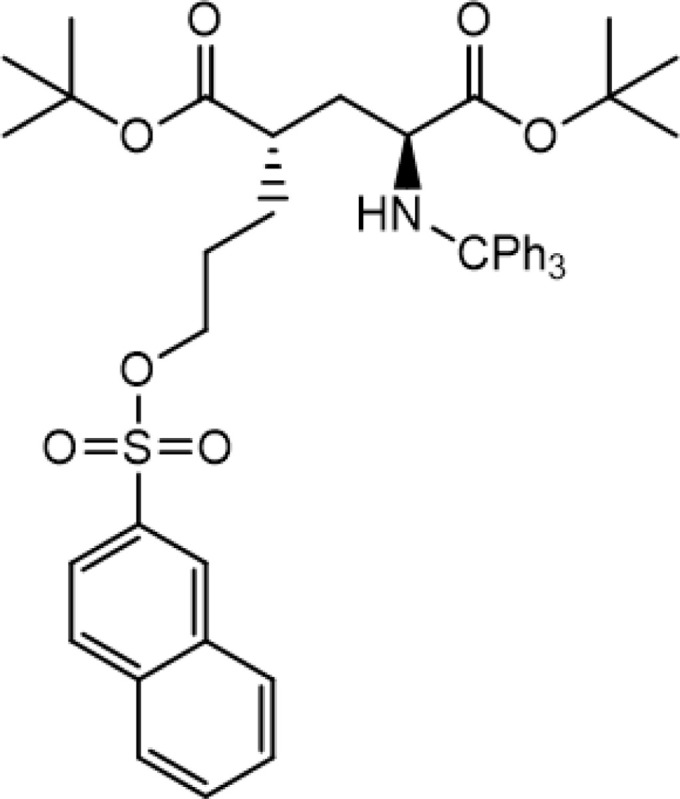
Chemical structure of the precursor to produce [^18^F]FSPG.

The automated radiosynthesis of [^18^F]FSPG using GE TRACERlab^™^ FXFN and FASTlab^™^ was performed inside the sterilized lead-shielded COMECER hot cell under cGMP condition. The production of [^18^F]FSPG on GE TRACERlab^™^ FXFN and FASTlab^™^ shares common consumables and reagents, in which the reference standard (*(4S)-4-(3-fluoropropyl)L-glutamate,* Prod. # 3194, Fig. 1) and cGMP-grade precursor (*di-tert-butyl (2S,4S)-2-(3-((naphthalen-2 ylsulfonyl)oxy)propyl)-4 (tritylamino) pentanedioate,* Prod. # 3193, Fig. 2) were purchased from ABX GmbH (Radeberg, Germany), as were the Cryptand-222 (Kryptofix^®^ [2.2.2]) (Prod. # 800) and the preconditioned QMA light cartridges (Prod. # K-920). The Oasis MCX Plus (60 μm LP, Part # 186003516) and Alumina N Plus long cartridges (Part # WAT020510) were acquired through Waters (Milford, MA). Supelclean ENVI-Carb SPE graphitized carbon (Cat# 57094) and 6 mL SPE tubes were obtained from Millipore Sigma (Burlington, MA). Acrodisc^®^ glass membrane filter and sterilized water for injection (SWI) were products of PALL Corp. and Hospira, respectively. All other references of ‘water’ refers to Milli-Q water (18 MΩ•cm) taken from a Millipore Milli-Q Integral 5 water purification system and were primarily used in quality control processes. Anhydrous acetonitrile (99.8%) used in the evaporation and labeling steps was from Sigma-Aldrich. Other reagents used in the production process of [^18^F]FSPG include sodium hydroxide (4M NaOH, RICCA), sulfuric acid (1M H_2_SO_4_, Fisher), ethanol (Pharmco), sodium phosphate dibasic dihydrate (Acros Organics); in addition, potassium carbonate and sodium chloride were purchased from Thermo Fisher.

The Kryptofix stock solution was prepared, in-house, by adding 94.0 ± 1.0 mg of Kryptofix (Cryptand-222) and 9.40 ± 0.10 mg of potassium carbonate in a glass container. The solid mixture was then dissolved with 5 mL of SWI and 5 mL of acetonitrile. The phosphate buffered solution used for final elution of [^18^F]FSPG contained 603 ± 10 mg of sodium chloride and 700 ± 10 mg of sodium phosphate dibasic dihydrate that were dissolved by 100 mL of SWI. All chemicals were used without further purification. Nitrogen and argon gas used primarily in drying and transferring of solutions were provided through Matheson Tri-gas. The automation synthesis on the TRACERlab^™^ FXFN and FASTlab^™^ modules were controlled by the TRACERLab FX software and the FASTlab Developer software, respectively.

### Automated synthesis of [^18^F]FSPG

#### Procedure Overview

[^18^F]Fluoride was produced by irradiating 2.5 mL of enriched [^18^O]H_2_O with 60 μAh beam current from the 16.5 MeV GE PETrace cyclotron. The [^18^F]Fluoride was separated from the [^18^O]H_2_O by trapping the [^18^F]Fluoride on the preconditioned QMA light cartridge. Following the trapping step, a solution of the Kryptofix stock solution was used to elute the [^18^F]Fluoride from the cartridge into the reaction vessel. The solution was heated to 120°C to remove the water and acetonitrile initially. Next, the precursor for the reaction, (2S,4S)di-*tert-butyl* 2-(3((naphthalene-2-ylsulfonyl)oxy)propyl)-4-(tritylamino)pentanedioate dissolved in anhydrous acetonitrile, was added to the reaction vessel. The reaction was heated at 105°C for 5 minutes in a closed reaction vessel to promote the substitution of the ^18^F for the sulfonate leaving group. Next, the 1M sulfuric acid solution was added and the reaction was heated at 105°C for 4 minutes to reveal the carboxylic acid groups. Hydrolysis occured as 4.0M sodium hydroxide was added and the reaction was heated to 70°C for a period of 5 minutes. The solution was then allowed to cool and a second batch of the 1M sulfuric acid was added to acidify the mixture. The reaction mixture was transferred onto the MCX cartridges connected in series. The cartridges were then washed with SWI to remove impurities. The [^18^F]FSPG was then desorbed from the MCX cartridges with the phosphate buffered solution. The [^18^F]FSPG was further purified by passing the eluent through an alumina N Sep-Pak cartridge and a SPE column packed with 1.5 g of ENVI-Carb graphite carbon to eliminate remaining [^18^F]fluoride and hydrophilic impurities. The desired product was finally passed through a 0.22 μm vented sterilizing filter into a sterile vial for QC sampling and dose dispensing.

#### GE TRACERlab^™^ FXFN Setup

**Figure 3:**
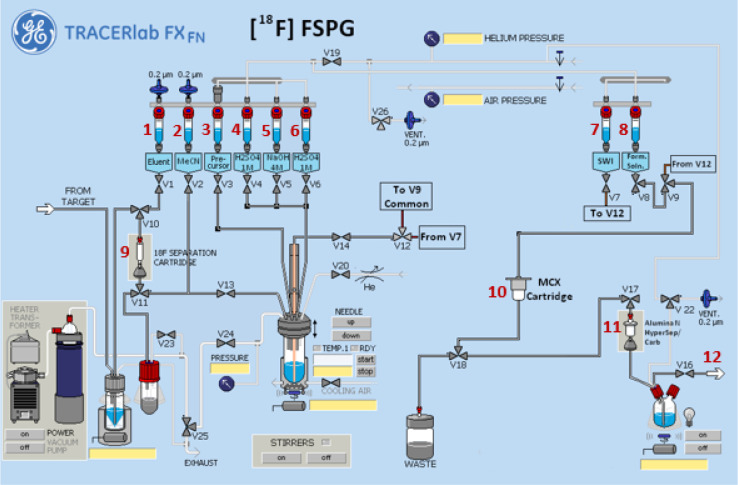
Schematic overview of the [^18^F]FSPG radiosynthesis on the GE TRACERlab^™^ FXFN module. The numbers denoted in RED, represented the designation of item and position of reagents and consumables indicated on Table 1.

**Figure 4:**
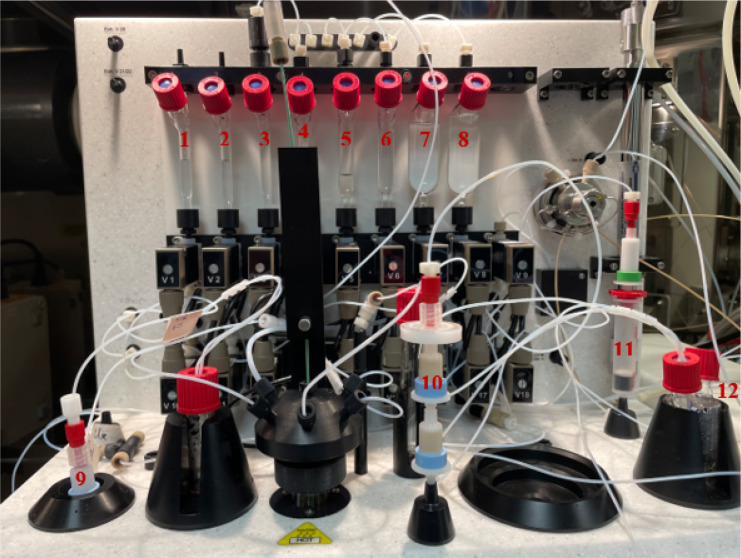
GE TRACERlab^™^ FXFN module set-up of reagents and cartridge/filter assembly on the for radiosynthesis of [^18^F]FSPG.

**Figure 5:**
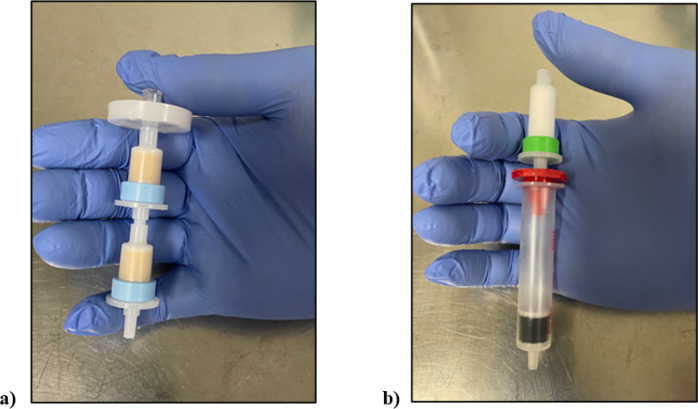
Assembling the Oasis^®^ MCX cartridge/Glass membrane filter set (a) and the Alumina N Cartridge/Superclean^™^ ENVI-Carb^™^ assembly (b). The assemblies were installed and activated only on the day of the synthesis.

Figure 3 and Fig. 4 illustrates the preparation setup for the radiosynthesis of [^18^F]FSPG for injection. Vials 1 through 8 were loaded with the respective reagents shown on Table 1. At position 9, a pre-conditioned QMA cartridge was installed and used as purchased. Two Oasis^®^ MCX cartridges were attached and conditioned with 10 mL of the formulated phosphate buffered saline, followed by 10 mL of air, then assembled to a single glass membrane filter, as demonstrated in Fig. 5a, and connected to the lines at position 10. To prepare the Alumina N Cartridge/Superclean^™^ ENVI-Carb^™^ assembly for the radiosynthesis, approximately 1.5 grams of ENVI-Carb resin was weighed and added to a 6 mL Supelco tube and a frit was packed in to secure the resin. The column was then sequentially conditioned with 10 mL of ethanol, followed by 10 mL of the formulated phosphate buffered saline, then dried with 10 mL of air. An Alumina N Plus Long SepPak cartridge was activated with 10 mL of SWI followed by 10 mL of air. The Alumina cartridge was then attached to the top of the ENVI-Carb column, as illustrated by Fig. 5b, and then assembled to the position 11 on the TRACERlab^™^ FXFN module. A final product vial was connected to the outgoing transfer line of the double-neck vial, as shown in position 12.

#### GE FASTlab^™^ Setup

**Figure 6:**
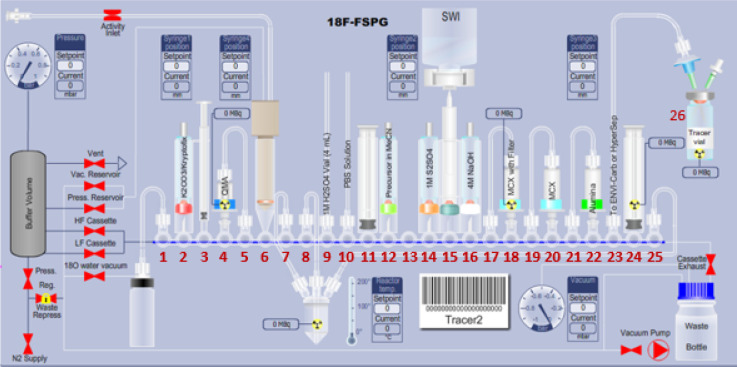
Schematic FASTlab cassette layouts for the radiosynthesis of [^18^F]FSPG. The numbers denoted in RED represent the designation of item and position of reagents and consumables indicated on Table 2.

**Figure 7:**
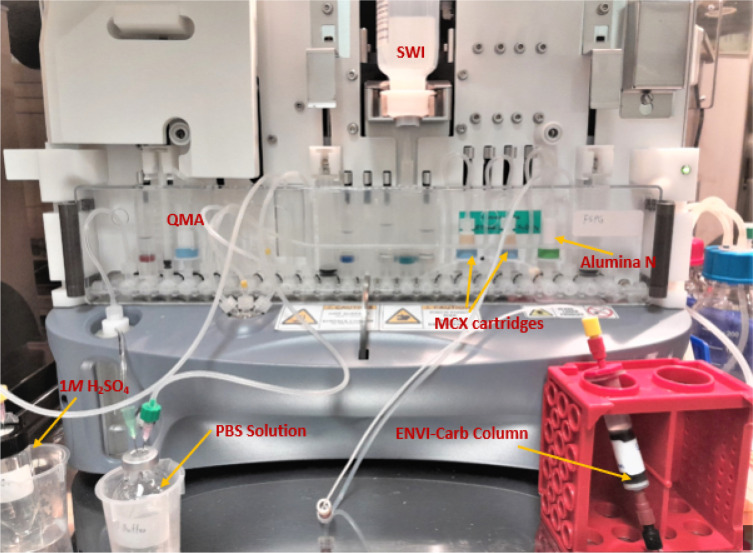
GE FASTlab^™^ set-up of reagents and cartridges on the for radiosynthesis of [^18^F]FSPG.

Figure 6 and Fig. 7 illustrates the preparation for the radiosynthesis of [^18^F]FSPG for injection using the cassette setup on the *FASTlab*^™^ module. As indicated on Table 2, reagent vials were prepared in 11 mm and 13 mm vials, crimped sealed and loaded to the cassette. The pre-conditioned QMA cartridge was installed and used as purchased. Similarly to the TRACERlab setup, the MCX and Alumina cartridges were conditioned with the same solution sequence and amount. Unlike the setup on the TRACERlab, the two Oasis^®^ MCX cartridges were placed in series in the cassette instead of stacking on top of each other. The FASTlab setup did not require the use of a glass membrane filter and the Alumina N Cartridge was integrated into the cassette as well. In this case, the Superclean^™^ ENVI-Carb^™^ column is the only purification column/ cartridge that was connected externally. For the radiosynthesis, approximately 1.5 grams of ENVI-Carb resin was weighed and added to a 6 mL Supelco tube and a frit was packed in to secure the resin. The column was then sequentially conditioned with 10 mL of ethanol, followed by 10 mL of the formulated phosphate buffered saline, then dried with 10 mL of air. The inlet of the column was connected to the tubing attached to the cassette position CP23. The outlet of the column was connected with the transfer line that would eventually delivered the purified [^18^F]FSPG for injection to the final product vial.

### Quality Control Method

The characterization of [^18^F]FSPG for injection was performed on the Agilent 1260 HPLC system equipped with a variable wavelength detector and Lablogic NaI radio-detector. The analytical method for determining the identity, chemical and radiochemical purity of the [^18^F]FSPG include the following conditions: The analytical peak separation flow rate was set to 1.5 mL/min through a Phenomenex Luna C-18(2) column (250 mm × 4.6 mm × 5 μm) and the UV detector @ 340 nm. Mobile Phase (A) 40 μM Disodium Phosphate in Water and Mobile Phase (B) Acetonitrile/Methanol/Water (45:45:10)(v/v), with gradient 0–4% B (2 min); 4–12% B (3 min); hold at 12% B (5 min); 12–60% B (5 min); 60–100% B (2 min); hold at 100% B (2 min); 100–0% B (1min); hold at 0% B (5 min). In this condition, [^18^F]FSPG was eluted at around 13 min.

**Figure 8:**
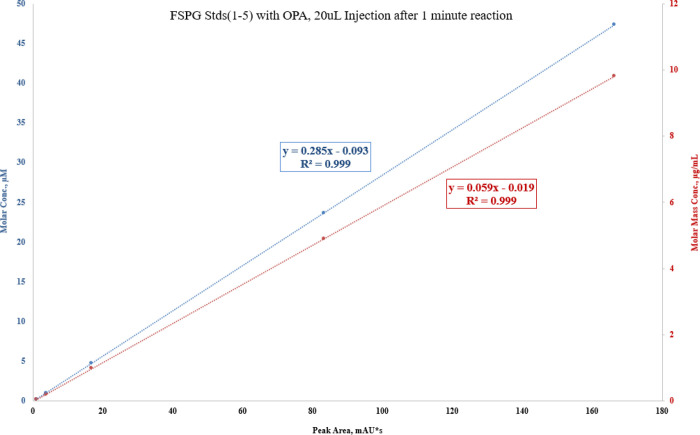
Five (5) point calibration of FSPG reference standard complexation with OPA reagent by HPLC analysis. The blue regression curve represents the molar concentration, whereas the red curve represents the mass concentration, measured against the peak area.

**Figure 9:**
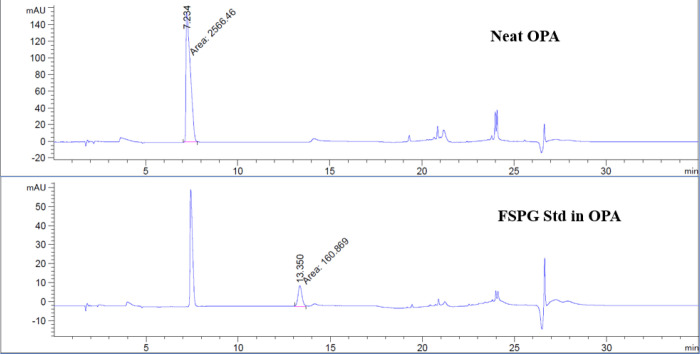
Illustration of UV chromatograms of FSPG reference standard after reaction with OPA reagent by HPLC analysis. Per the method described previously (in Quality Control section), the OPA peak elutes around 7 minutes and FSPG standard elutes at the 13 minutes region.

**Figure 10:**
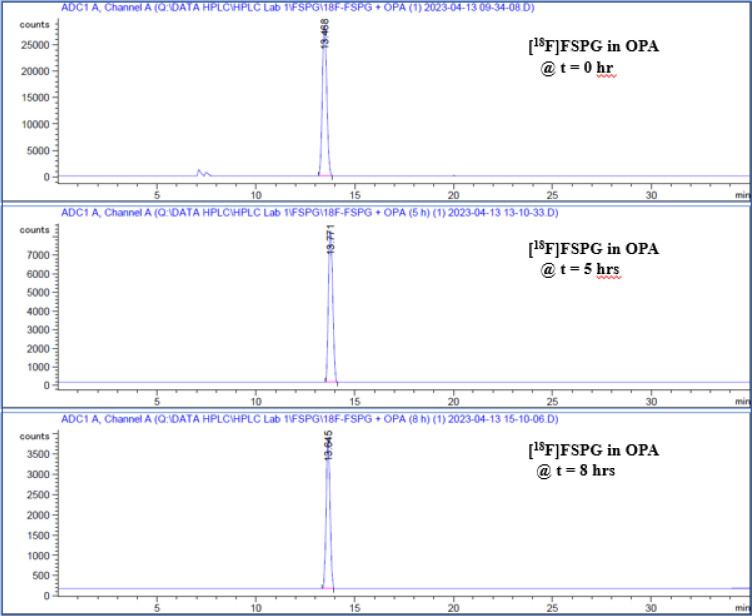
Radio-HPLC chromatograms of [^18^F]FSPG after reaction with OPA reagent. Per the method described previously (in Quality Control section), [^18^F]FSPG peak elutes at the 13 minutes region. The indicated chromatograms verified that [^18^F]FSPG remained stable for 8 hours after final batch formulation.

To prepare sample for analysis, 80 μL of FSPG reference standard was first complexed with an 20 μL of OPA reagent. The mixture of the reference standard OPA reagent was vortexed and allowed to react for 1 minute, then injected to the HPLC. Similarly, complexation of [^18^F]FSPG with the OPA reagent, in 4:1 ratio, was injected and compared to the previously injected reference standard-OPA complex. Figures 8, 9 and 10 illustrated the data results by HPLC analysis for calibration of FSPG reference standard, identification of OPA and cold FSPG standard, and identity and stability of [^18^F]FSPG for injection, respectively.

The residual solvents in [^18^F]FSPG for injection was determined by Agilent GC system with FID, equipped with GC Column DB200 (30 m × 0.250 mm, 0.50 μm stationary phase thickness). The radionuclidic identity and purity, residual Kryptofix^®^ [2.2.2], bacterial endotoxin, sterility. appearance, pH, and filter tests were performed under the USP < 823 > guidelines.

## Results

### Automated synthesis of [^18^F]FSPG

The production of [^18^F]FSPG was completed within 45 min in the TRACERlab^™^ FXFN including the trapping of [^18^F]fluoride from the cyclotron to the QMA cartridge on the synthesizer. The results of [^18^F]FSPG synthesis using the TRACERlab are shown in Table 3. The average non-decay-corrected radiochemical yield was 22.56 ± 0.97% with radiochemical purity of 96.31 ± 0.62% when less than 37 GBq of the starting activity and 6 mg of the precursor were applied. However, dramatical reduction in radiochemical yield and slightly lower radiochemical purity were observed when larger starting activity was used. This limit could further be resolved by adding additional amounts of the precursor and ENVI-Carb graphitized carbon for the reaction and final purification process. Because our ultimate goal was to facilitate the centralized production of [^18^F]FSPG for clinical use, we translated these findings to FASTlab^™^, a cassette-based module, for easier adoption by other radiopharmaceutical manufacturing facilities.

Similar to automated synthesis performed in the TRACERlab, the production of [^18^F]FSPG was completed within 42 min in the FASTlab. The results of [^18^F]FSPG automated synthesis using a single-use cassette with 12 mg of the precursor and 1.5 g of ENVI-Carb graphitized carbon are shown in Table 4. While a consistently high radiochemical purity (95.96 ± 0.70%) was maintained, the average radiochemical yields was also observed to be significantly higher than from the TRACERlab counterpart, 30.82 ± 1.60% vs. 22.56 ± 0.97%, *p* < 0.0001. The much improved radiochemical yields from the FASTlab, in addition to changes previously mentioned, can also be attributed to the compact design of the reactor and more efficient built-in vacuum system in the module compared to the counterpart TRACERlab.

### Quality Control of [^18^F]FSPG for Injection

Radio-HPLC analysis of the produced [^18^F]FSPG for injection showed > 95% radiochemical purity. Further analysis after 8h confirmed that the product remained stable (Fig. 10). The molar activity calculated from the activity yields at EOS and FSPG was in the range of 100–292.3 GBq/μmol. The residual ethanol and acetonitrile concentrations were less than 0.2% and 0.005% (v/v), respectively. The pH was 6.5 to 7.5 and the bubble point was 70 to 75 psi. In addition, the sterility, pyrogenicity, and all other quality control tests passed specifications based on the USP < 823 > guidelines.

## Discussion

In this study, our work was undertaken to support a variety of clinical trials leveraging [^18^F]FSPG PET. We have achieved the cGMP-compliant automated production of [^18^F]FSPG with optimal synthesis procedure for both GE TRACERlab^™^ FXFN and FASTlab^™^ synthesizer. While several commercially available modules for [^18^F]FSPG production have been previously published (Brown et al., 2023; Edwards et al., 2021; Kavanaugh et al., 2016; Koglin et al., 2011; McCormick et al., 2019; Mittra et al., 2016; Shih et al., 2020), our method has consistently produced [^18^F]FSPG at high radiochemical purity and high molar activity. In particular, our adaptation of the SPE purification method was elegantly simplified in setup and with minimal processing time, making it attractive for centralized and large-scale production of [^18^F]FSPG at a manufacturing facility. During our production process, the sulfuric acid and sodium hydroxide were added to the crude reaction mixture to remove trityl and t-butyl protecting groups following radio-fluorination. The solution was then acidified by adding the sulfuric acid a second time prior to passing through the MCX cartridges. [^18^F]FSPG and the non-reacted precursor were trapped on the cartridges whereas most [^18^F]fluoride and water-soluble impurities were eliminated by the MCX, a cation exchange cartridge. Once the phosphate buffered solution was passed through the cartridges, the ion-exchange retention mechanism was terminated because the trapped compounds become negatively charged or unionized. However, due to the reverse-phase characteristic of the MCX cartridge sorbent, most of the non-reacted precursor was retained as a result of its lipophilic nature while [^18^F]FSPG was found to be well-eluted. The eluted crude [^18^F]FSPG solution was further purified by an Alumina N cartridge and SPE column containing ENVI-Carb graphitized carbon to remove remaining [^18^F]fluoride and the impurities from the side reactions, respectively. Compared to the previous reports that require 83 min to produce [^18^F]FSPG in the TRACERlab^™^ and 60 min in the FASTlab^™^ (Edwards et al., 2021; Shih et al., 2020), our production time was greatly reduced to within 45 min in both modules. More importantly, all commercially available cartridges and resins can be used directly without any modifications in our production process, presenting it ideal protocol for cGMP manufacturing of [^18^F]FSPG for injection.

## Conclusions

We have developed highly-reliable and simplified automated methods for the routine production of [^18^F]FSPG with high activity concentration and radiochemical purity using two commercially available synthesizers. While the clinical trials using [^18^F]FSPG PET are currently underway, the automated approaches reported herein will accelerate the clinical adoption of this radiotracer and enable centralized and large-scale production of [^18^F]FSPG.

## Figures and Tables

**Table 1:** Material and reagent list used in the radiosynthesis of [^18^F]FSPG *via* TRACERlab^™^ FXFN module

^a^ Item #	Reagents or consumables
**1**	Potassium Carbonate/Kryptofix QMA Elution Solution, 0.6 mL
**2**	Anhydrous Acetonitrile, 0.6 mL
**3**	FSPG precursor, 6 mg dissolved in 1.0 mL anhydrous acetonitrile
**4**	1M H_2_SO_4_, 2.0 mL
**5**	4M NaOH, 1.5 mL
**6**	1M H_2_SO_4_, 4.0 mL
**7**	Sterile water for injection (SWI), 15.0 mL
**8**	^b^ Phosphate buffer solution, 20.0 mL
**9**	Pre-conditioned QMA light SepPak cartridge, 1 cartridge
**10**	^c^ Oasis^®^ MCX cartridge/Glass membrane filter assembly, 1 set
**11**	^d^ Alumina N Plus Long Cartridge/Superclean^™^ ENVI-Carb^™^ assembly, 1 set
**12**	Final Product Vial, 1 vial

a *Items designation and position are indicated in* Fig. 3.

b *Formulation of Phosphate buffer solution is specified in the materials and methods general section.*

c *Illustration of the Oasis^®^ MCX cartridge/Glass membrane filter assembly is shown in* Fig. 5a.

d *Illustration of the Alumina N Cartridge/Superclean ^™^ ENVI-Carb ^™^ assembly is shown in* Fig. 5b.

**Table 2:** Cassette position and materials/reagents list used in the radiosynthesis of [^18^F]FSPG *via* FASTlab^™^ module

^a^ Cassette position (CP)	Reagents or consumables
**1**	Short tubing to ^18^O water collection vial
**2**	Potassium Carbonate/Kryptofix QMA Elution Solution, (11 mm Vial, 0.8 mL)
**3**	Left Hand Syringe, Syringe 1
**4**	QMA light SepPak cartridge, 1 pre-condition cartridge
**5**	Short tubing to QMA light SepPak cartridge at CP4
**6**	^18^F Inlet
**7**	Short tubing to reactor side port
**8**	Short tubing to reactor center port
**9**	1M H_2_SO_4_, long tubing connected to external 4.0 mL 1M H_2_SO_4_ vial
**10**	^b^ Phosphate Buffered Saline (PBS), long tubing connected to external 20 mL PBS vial
**11**	Middle Syringe (MS), Syringe 2
**12**	FSPG precursor, (11 mm Vial, 6–12 mg dissolved in 1.3 mL anhydrous acetonitrile)
**13**	Unused
**14**	1M H_2_SO_4_, (13 mm Vial, 2.2 mL)
**15**	Water Spike/water bag, 100 mL SWI bag
**16**	4M NaOH (13 mm Vial, 1.7 mL)
**17**	Short tubing to Oasis MCX cartridge at CP18
**18**	Oasis^®^ MCX cartridge, 1 cartridge
**19**	Short tubing to Oasis MCX cartridge at CP20
**20**	Oasis^®^ MCX cartridge, 1 cartridge
**21**	Short tubing to Alumina N Plus Long SepPak cartridge at CP22
**22**	Alumina N Plus Long SepPak Cartridge, 1 cartridge
**23**	Connecting tube to Superclean^™^ ENVI-Carb^™^ packed column, 1 set
**24**	Right Hand Syringe (RHS), Syringe 3
**25**	Long tubing to reactor side port
**26**	Final Product Vial, 1 vial

a *Items designation and position are indicated in* Fig. 6.

b *Formulation of Phosphate buffer solution is specified in the materials and methods general section.*

**Table 3.** Radiosynthesis of [^18^F]FSPG *via* TRACERlab^™^ FXFN module

Entry	Starting Activity (G8q)	Precursor Amunt (mg)	Total Synthetic Time (min)	Activity of the Obtained [^l8^F]FSPG (GBq)	Amount of ENVI-Carb graphitized carbon (g)	Radiochemical Purity	Non-decay-corrected Radiochemical Yield
1	14.80	6	45	3.19	0.5	96.73%	21.60%
2	24.05	6	45	5.77	0.5	96.79%	23.99%
3	26.64	6	45	6.07	0.5	95.81%	22.79%
4	33.56	6	45	7.62	0.5	95.48%	22.71%
5	37.00	6	45	8.03	0.5	96.72%	21.70%
6	92.50	6	45	7.51	0.5	93.29%	8.12%
7	64.01	12	45	12.65	1.5	98.60%	19.76%
8	64.75	12	45	12.77	1.5	95.99%	19.72%
9	85.10	12	45	15.80	1.5	95.03%	18.57%

**Table 4.** Radiosynthesis of [^18^F]FSPG *via* FASTlab^™^ module

Entry	Starting Activity (G8q)	Precursor Amunt (mg)	Total Synthetic Time (min)	Activity of the Obtained [^18^F]FSPG (GBq)	Amount of ENVI-Carb graphitized carbon (g)	Radiochemical Purity	Non-decay-corrected Radiochemical Yield
1	63.68	12	42	19.31	1.5	95.50%	30.32%
2	68.97	12	42	22.46	1.5	95.87%	32.65%
3	59.68	12	42	17.24	1.5	96.98%	28.89%
4	62.42	12	42	19.61	1.5	95.50%	31.42%

## Data Availability

All data generated or analyzed during this study are included in this manuscript.

## References

[1] Baek S., Choi C. M., Ahn S. H., Lee J. W., Gong G., Ryu J. S., . . . Moon D. H. (2012). Exploratory clinical trial of (4S)-4-(3-[^18^F]fluoropropyl)-L-glutamate for imaging xC- transporter using positron emission tomography in patients with non-small cell lung or breast cancer. Clin Cancer Res, 18(19), 5427–5437. doi:10.1158/1078-0432.CCR-12-0214 22893629

[2] Brown G., Soloviev D., & Lewis D. Y. (2023). Radiosynthesis and Analysis of (S)-4-(3-[^18^F]Fluoropropyl)-L-Glutamic Acid. Mol Imaging Biol, 25(3), 586–595. doi:10.1007/s11307-022-01793-3 36525163 PMC10172245

[3] Carbone M., Harbour J. W., Brugarolas J., Bononi A., Pagano I., Dey A., . . . Gaudino G. (2020). Biological Mechanisms and Clinical Significance of BAP1 Mutations in Human Cancer. Cancer Discov, 10(8), 1103–1120. doi:10.1158/2159-8290.Cd-19-1220 32690542 PMC8006752

[4] Combs J. A., & DeNicola G. M. (2019). The Non-Essential Amino Acid Cysteine Becomes Essential for Tumor Proliferation and Survival. Cancers, 11(5), 678. 31100816 10.3390/cancers11050678 PMC6562400

[5] Edwards R., Greenwood H. E., McRobbie G., Khan I., & Witney T. H. (2021). Robust and Facile Automated Radiosynthesis of [^18^F]FSPG on the GE FASTlab. Mol Imaging Biol, 23(6), 854–864. doi:10.1007/s11307-021-01609-w 34013395 PMC8578107

[6] Han A., Purwin T. J., & Aplin A. E. (2021). Roles of the BAP1 Tumor Suppressor in Cell Metabolism. Cancer Research, 81(11), 2807–2814. doi:10.1158/0008-5472.Can-20-3430 33446574 PMC8178170

[7] Kavanaugh G., Williams J., Morris A. S., Nickels M. L., Walker R., Koglin N., . . . Manning H. C. (2016). Utility of [^18^F]FSPG PET to Image Hepatocellular Carcinoma: First Clinical Evaluation in a US Population. Mol Imaging Biol, 18(6), 924–934. doi:10.1007/s11307-016-1007-0 27677886 PMC5641676

[8] Koglin N., Mueller A., Berndt M., Schmitt-Willich H., Toschi L., Stephens A. W., . . . Dinkelborg L. M. (2011). Specific PET Imaging of xC− Transporter Activity Using a ^18^F-Labeled Glutamate Derivative Reveals a Dominant Pathway in Tumor Metabolism. Clinical Cancer Research, 17(18), 6000–6011. doi:10.1158/1078-0432.Ccr-11-0687 21750203

[9] Koppula P., Zhang Y., Zhuang L., & Gan B. (2018). Amino acid transporter SLC7A11/xCT at the crossroads of regulating redox homeostasis and nutrient dependency of cancer. Cancer Commun (Lond), 38(1), 12. doi:10.1186/s40880-018-0288-x 29764521 PMC5993148

[10] Lei G., Zhang Y., Koppula P., Liu X., Zhang J., Lin S. H., . . . Gan B. (2020). The role of ferroptosis in ionizing radiation-induced cell death and tumor suppression. Cell Research, 30(2), 146–162. doi:10.1038/s41422-019-0263-3 31949285 PMC7015061

[11] Lewerenz J., Hewett S. J., Huang Y., Lambros M., Gout P. W., Kalivas P. W., . . . Maher P. (2013). The cystine/glutamate antiporter system x(c)(−) in health and disease: from molecular mechanisms to novel therapeutic opportunities. Antioxid Redox Signal, 18(5), 522–555. doi:10.1089/ars.2011.4391 22667998 PMC3545354

[12] Lo M., Ling V., Wang Y. Z., & Gout P. W. (2008). The xc- cystine/glutamate antiporter: a mediator of pancreatic cancer growth with a role in drug resistance. British journal of cancer, 99(3), 464–472. doi:10.1038/sj.bjc.6604485 18648370 PMC2527809

[13] Magarik M. A., Walker R. C., Gilbert J., Manning H. C., & Massion P. P. (2018). Intracardiac Metastases Detected by ^18^F-FSPG PET/CT. Clin Nucl Med, 43(1), 28–30. doi:10.1097/RLU.0000000000001883 29076915 PMC5716866

[14] McCormick P. N., Greenwood H. E., Glaser M., Maddocks O. D. K., Gendron T., Sander K., . . . Witney T. H. (2019). Assessment of Tumor Redox Status through (S)-4-(3-[^18^F]fluoropropyl)-L-Glutamic Acid PET Imaging of System x(c) (−) Activity. Cancer Res, 79(4), 853–863. doi:10.1158/0008-5472.Can-18-2634 30401715 PMC6379064

[15] Mittra E. S., Koglin N., Mosci C., Kumar M., Hoehne A., Keu K. V., . . . Gambhir S. S. (2016). Pilot Preclinical and Clinical Evaluation of (4S)-4-(3-[^18^F]Fluoropropyl)-L-Glutamate (^18^F-FSPG) for PET/CT Imaging of Intracranial Malignancies. PLoS One, 11(2), e0148628. doi:10.1371/journal.pone.0148628 26890637 PMC4758607

[16] Mosci C., Kumar M., Smolarz K., Koglin N., Stephens A. W., Schwaiger M., . . . Mittra E. S. (2016). Characterization of Physiologic ^18^F-FSPG Uptake in Healthy Volunteers. Radiology, 279(3), 898–905. doi:10.1148/radiol.2015142000 26785040

[17] Murali R., Wiesner T., & Scolyer R. A. (2013). Tumours associated with BAP1 mutations. Pathology, 45(2), 116–126. doi:10.1097/PAT.0b013e32835d0efb 23277170

[18] Paez R., Shah C., Cords A. J., Muterspaugh A., Helton J. E., Antic S., . . . Massion P. P. (2022). ^18^F-FSPG PET imaging for the evaluation of indeterminate pulmonary nodules. PLoS One, 17(3), e0265427. doi:10.1371/journal.pone.0265427 35294486 PMC8926263

[19] Park S., Hatami N., Rutledge O., Koglin N., Loo B., Fan A., & Mittra E. (2017). Pilot study of ^18^F-FSPG vs ^18^F-FDG PET imaging for response assessment in cancer. Journal of Nuclear Medicine, 58(supplement 1), 118.

[20] Park S. Y., Mosci C., Kumar M., Wardak M., Koglin N., Bullich S., . . . Mittra E. S. (2020). Initial evaluation of (4S)-4-(3-[^18^F]fluoropropyl)-l-glutamate (FSPG) PET/CT imaging in patients with head and neck cancer, colorectal cancer, or non-Hodgkin lymphoma. EJNMMI Research, 10(1), 100. doi:10.1186/s13550-020-00678-2 32857284 PMC7455665

[21] Shih K. T., Huang Y. Y., Yang C. Y., Cheng M. F., Tien Y. W., Shiue C. Y., . . . Hsin L. W. (2020). Synthesis and analysis of 4-(3-fluoropropyl)-glutamic acid stereoisomers to determine the stereochemical purity of (4S)-4-(3-[^18^F]fluoropropyl)-L-glutamic acid ([^18^F]FSPG) for clinical use. PLOS ONE, 15(12), e0243831. doi:10.1371/journal.pone.0243831 33315962 PMC7735610

[22] Smolarz K., Krause B. J., Graner F. P., Wagner F. M., Hultsch C., Bacher-Stier C., . . . Schwaiger M. (2013). (S)-4-(3–18F-fluoropropyl)-L-glutamic acid: an ^18^F-labeled tumor-specific probe for PET/CT imaging--dosimetry. J Nucl Med, 54(6), 861–866. doi:10.2967/jnumed.112.112581 23568366

[23] Wardak M., Sonni I., Fan A. P., Minamimoto R., Jamali M., Hatami N., . . . Mittra E. S. (2022). 18F-FSPG PET/CT Imaging of System XC− Transporter Activity in Patients with Primary and Metastatic Brain Tumors. Radiology, 303(3), 620–631. doi:10.1148/radiol.203296 35191738 PMC9131170

[24] Yan Y. C., Meng G. X., Ding Z. N., Liu Y. F., Chen Z. Q., Yan L. J., . . . Li T. (2022). Somatic mutation and expression of BAP1 in hepatocellular carcinoma: an indicator for ferroptosis and immune checkpoint inhibitor therapies. J Cancer, 13(1), 88–101. doi:10.7150/jca.65574 34976173 PMC8692694

[25] Zhang Y., Shi J., Liu X., Feng L., Gong Z., Koppula P., . . . Gan B. (2018). BAP1 links metabolic regulation of ferroptosis to tumour suppression. Nature Cell Biology, 20(10), 1181–1192. doi:10.1038/s41556-018-0178-0 30202049 PMC6170713

